# Targeted quantitative profiling of metabolites and gene transcripts associated with 4-aminobutyrate (GABA) in apple fruit stored under multiple abiotic stresses

**DOI:** 10.1038/s41438-018-0069-3

**Published:** 2018-12-01

**Authors:** Carolyne J. Brikis, Adel Zarei, Greta Z. Chiu, Kristen L. Deyman, Jingyun Liu, Christopher P. Trobacher, Gordon J. Hoover, Sanjeena Subedi, Jennifer R. DeEll, Gale G. Bozzo, Barry J. Shelp

**Affiliations:** 1 Department of Plant Agriculture, University of Guelph, Guelph, Ontario N1G 2W1 Canada; 2 Department of Mathematical Sciences, Binghamton University, Binghamton, NY 13902 USA; 3 Ontario Ministry of Agriculture, Food and Rural Affairs, Box 587, 1283 Blueline Rd. at Highway 3, Simcoe, Ontario N3Y 4N5 Canada

## Abstract

4-Aminobutyrate accumulates in plants under abiotic stress. Here, targeted quantitative profiling of metabolites and transcripts was conducted to monitor glutamate- and polyamine-derived 4-aminobutyrate production and its subsequent catabolism to succinate or 4-hydroxybutyrate in apple (*Malus* x *domestica* Borkh.) fruit stored at 0 °C with 2.5 kPa O_2_ and 0.03 or 5 kPa CO_2_ for 16 weeks. Low-temperature-induced protein hydrolysis appeared to be responsible for the enhanced availability of amino acids during early storage, and the resulting higher glutamate level stimulated 4-aminobutyrate levels more than polyamines. Elevated CO_2_ increased the levels of polyamines, as well as succinate and 4-hydroxybutyrate, during early storage, and 4-aminobutyrate and 4-hydroxybutyrate over the longer term. Expression of all of the genes likely involved in 4-aminobutyrate metabolism from glutamate/polyamines to succinate/4-hydroxybutyrate was induced in a co-ordinated manner. CO_2_-regulated expression of apple *GLUTAMATE DECARBOXYLASE 2*, *AMINE OXIDASE 1*, *ALDEHYDE DEHYDROGENASE 10A8* and *POLYAMINE OXIDASE 2* was evident with longer term storage. Evidence suggested that respiratory activities were restricted by the elevated CO_2_/O_2_ environment, and that decreasing NAD^+^ availability and increasing NADPH and NADPH/NADP^+^, respectively, played key roles in the regulation of succinate and 4-hydroxybutyate accumulation. Together, these findings suggest that both transcriptional and biochemical mechanisms are associated with 4-aminobutyrate and 4-hydroxybutyrate metabolism in apple fruit stored under multiple abiotic stresses.

## Fruit storage: Biochemical effects in apples

The low temperature, reduced oxygen and elevated carbon dioxide conditions under which harvested apples are often stored to delay ripening causes changes in gene activity and metabolism associated with the amino acid 4-aminobutyrate. The effect of these storage conditions, which expose the fruit to a state known as abiotic stress, has not previously been studied in detail. Researchers in Canada and the US, led by Barry Shelp at the University of Guelph in Ontario, studied Empire apples stored in the typical artificial storage conditions for up to 16 weeks. They identified several genes whose activity increased during the study period. These changes were accompanied by increased breakdown of proteins in the fruit, the accumulation of 4-aminobutyrate and altered levels of several other related biochemicals. Understanding these biochemical responses may help optimize fruit storage procedures.

## Introduction

The non-protein amino acid 4-aminobutyrate (GABA) is derived from glutamate in plants exposed to various abiotic stresses via activity of the enzyme glutamate decarboxylase (GAD), which can be activated by Ca^2+^*/*calmodulin or stimulated by cytosolic acidification^1^. In turn, GABA is converted to succinic semialdehyde (SSA) via GABA transaminase (GABA-T) and then to succinate via NAD^+^-dependent succinic semialdehyde dehydrogenase (SSADH) or to 4-hydroxybutyrate (GHB) via NADPH-dependent glyoxylate/succinic semialdehyde reductase (GLYR)^1–3^. Much less attention has been paid to the derivation of GABA from polyamines^1,4^. This can occur by the terminal catabolism of putrescine (Put) to 4-aminobutanal/Δ^1^-pyrroline via O_2_-dependent copper-containing amine oxidases (AO) and spermidine to 4-aminobutanal via FAD-dependent polyamine oxidases (PAO)^1,4,5^, or the non-enzymatic decarboxylation of proline to pyrrolidin-1-yl, which is easily converted to 4-aminobutanal^6^. In turn, 4-aminobutanal can be converted to GABA via members of the aldehyde dehydrogenase (ALDH)10 family (i.e., NAD^+^-dependent ALDH10A8 and ALDH10A9)^7,8^.

In developed countries apple (*Malus* × *domestica* Borkh.) fruit are stored under controlled-atmosphere (CA) conditions (i.e., low O_2_ and elevated CO_2_) at low temperature to delay ripening^9,10^. Low temperature, low O_2_ or elevated CO_2_ have been associated with GABA accumulation in many plant systems^1,11–15^ and limited O_2_ availability has been associated with changes in redox balance^16,17^, which may in turn influence the activities of AO, ALDH10A, SSADH and GLYR^1,18,19^. If so, low-O_2_ and elevated-CO_2_ storage of apple fruit at low temperature could inhibit the production of GABA from polyamines and divert glutamate-derived GABA catabolism to GHB^20^.

Many of the experimental approaches that have been used to investigate the pathways associated with GABA metabolism in plants subjected to abiotic stress^1,20^ are not suited or readily adapted to study their relative importance in apples. For example, mutants are not available, and amine oxidase inhibitors and radiolabelled precursors cannot be supplied to intact fruit without perturbing the internal gaseous environment. Untargeted metabolomics and enzymatic approaches could provide a global view of the metabolism in the stressed fruit; however, the incomplete profiling of important GABA pathway metabolites and the presence of multiple enzyme forms, respectively, could severely limit the interpretation of these results. In the present study, we utilized targeted quantitative profiling of metabolites and gene transcripts in cultivar ‘Empire’ apple fruit to monitor GABA metabolism during 16 weeks of postharvest storage under multiple abiotic stress conditions (0 °C, 2.5 kPa O_2_ and 0.03 or 5 kPa CO_2_). Intact fruit were analyzed to eliminate any impact of the mechanical stress that would occur during separation of the peel and flesh^15,21,22^. Linear and multivariate correlation analyses were used to identify if any metabolites and gene transcripts were specifically associated with low-temperature/low-O_2_ conditions in the absence or presence of elevated CO_2_.

## Materials and methods

### Controlled-atmosphere storage

The harvest, low-temperature CA storage, and quality assessment in 2009 of the apple (*Malus* x *domestica* Borkh. cultivar ‘Empire’) fruit have been described elsewhere^9^. Here we chose the fruit collected from orchard 2. Immediately prior to storage, eight apples were randomly sampled from the bulk apples for assessment of physiological disorders. Four apples were also sampled and rapidly frozen in liquid N_2_ for assessment of metabolite and gene transcript levels. The remaining apples were stored at 0 °C in two CA rooms (i.e., treatment blocks). Briefly, within each CA room, two random duplicate chambers were supplied with either 0.03 (control) or 5 (CO_2_-treated) kPa CO_2_ in combination with 2.5 kPa O_2_ (i.e., a split-plot design) for 16 wk. At several times during storage, eight apples were randomly sampled from each treatment replicate for assessment of physiological disorders, and four apples (i.e., subsamples) were randomly sampled from each treatment replicate and rapidly frozen in liquid N_2_. All frozen apples were stored for several months before being individually pulverized to powder using an arbor press, taking care to ensure that thawing did not occur, before being stored at −80 °C. None of the freshly collected or stored fruit showed signs of flesh browning or senescent breakdown^21^, but external CO_2_ injury (i.e., bronze- to brown-colored, rough uneven lesion with sunken areas on the peel) was evident on fruit receiving 5 kPa CO_2_; the incidence was 37, 51, 70 and 85%, respectively, after 2, 4, 8 and 16 weeks of storage^9^.

### Extraction and analysis of metabolites

The frozen apple fruit powders were extracted within 1–3 years of harvest using various protocols, depending on the metabolites under consideration. The levels of various amino acids, including GABA, and the free forms of putrescine, spermidine and spermine were determined by reverse-phase high performance liquid chromatography as described elsewhere^14,23^. Detailed protocols for the extraction and GC-MS determination of GHB, and the enzymatic determination of succinate and pyridine dinucleotides are given in the Supplementary Information Materials and Methods S1.

### RNA extraction and cDNA synthesis

RNA was isolated from the frozen apple fruit powders for three treatment blocks within a year of harvest essentially as described elsewhere^15^. RNA integrity was verified using formaldehyde RNA gel electrophoresis. RNA (1 µg) was treated with DNAase I using the Turbo DNA-free kit (Applied Biosystems) according to the manufacturer’s protocol. For first strand cDNA synthesis, 10–100 ng total RNA was incubated with oligo(dT)20 and Superscript III RT (Invitrogen) at 50 °C, followed by 55 °C for 30 min.

### Identification of apple genes

The apple genes for three *GAD*s, two *GABA-T*s, two *GLYR*s, five *AO*s and two *ALDH10A*s have been reported elsewhere^5,7,15,24^. Methods for identifying the putative apple *SSADH*, *ALANINE TRANSAMINASE* and *POLYAMINE OXIDASE* genes are described in Supplementary Information Materials and Methods S2.

### Quantitative real-time PCR

Primers used for quantitative polymerase chain reaction (qPCR) were designed using Primer Express 3 software (Applied Biosystems) with the following default conditions: 60 °C primer melting temperature; 50–80 bp amplicon length; and, 40–60% primer GC content. The list of primers used here is provided in Supplementary Information Table S3. It was not possible to design primers that enabled separate monitoring of the two distinct apple *GABA-T* genes^15^. Quantitative PCR was performed in a 96-well plate iQ5 Multicolor Real-Time PCR Detection System (BioRad) as previously described^5^. Dissociation curve analysis was performed after 40 cycles of qPCR to ensure the presence of a single PCR product. Efficiency of the primer pairs ranged from 90 to 105%. The data were analyzed and relative expression calculated using the 2^−ΔCT^ method^25^. The expression of each target gene was normalized to the housekeeping apple *ELONGATION FACTOR-1α* (*EF-1α*) gene (MD0000294265)^26,27^. Each treatment replicate was analyzed in duplicate.

### Statistical analysis

The data were analyzed as a completely randomized design of two blocks (i.e., replicate CA rooms) with a split-plot design using ANOVAs (Proc Mixed method of SAS^®^ software^28^). Since there was no block effect for the incidence of external and internal disorders, time course data for levels of metabolites and transcripts are presented as means of four and three treatment replicates, respectively. Four apples were subsampled periodically from each treatment replicate; these were considered as repeated measures. Assumptions of randomness, homogeneity, and independence of errors were confirmed using plots of residuals, as well as a Shapiro–Wilk test for normality. Treatment means were compared within and across weeks using the Tukey’s Least Significance Difference method for multiple comparisons at the 95% confidence level.

The relationships among metabolites and transcripts were assessed by Pearson’s correlation test using R^29^ and corrected for false discovery rate^30^. The relationships among metabolites and transcripts was also assessed by principal component analysis (PCA) using R^29^. Replicates with missing subsample values for a variable/treatment were removed from the analysis. In some cases, the data set for each variable was not normally distributed when expressed on their original scale; therefore, the Shapiro–Wilk test for normality was performed on each variable individually. If a variable deviated from normality, boxcox transformation was used to identify transformed variables that approximate normality. Then the variables were scaled to a mean of 0 and a variance of 1 prior to PCA.

## Results

### Levels and ratios of pyridine dinucleotides

**Fig. 1:**
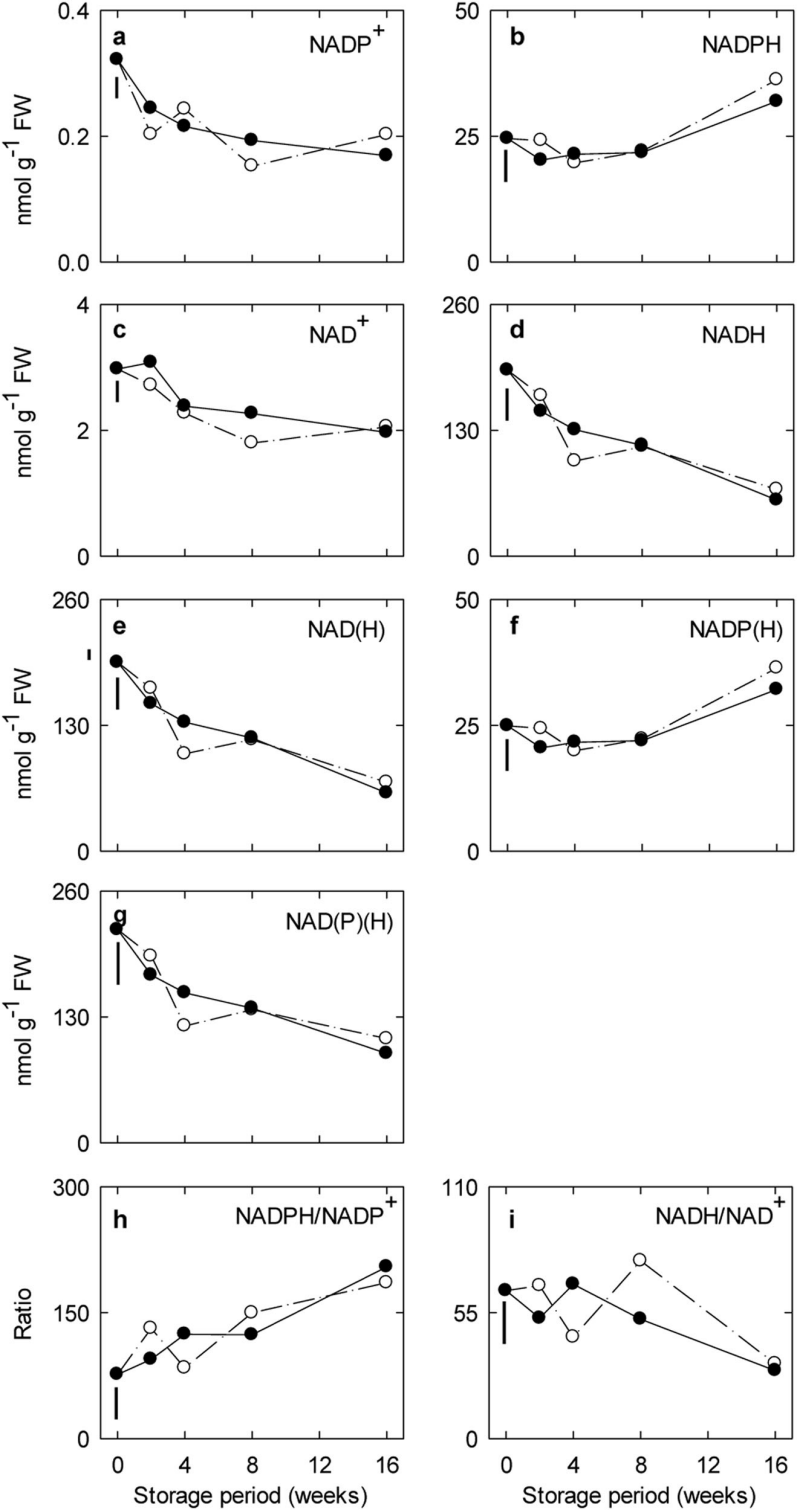
Impact of elevated CO_2_ on the pyridine dinucleotide status in ‘Empire’ apple fruit under low-temperature/low-O_2_ storage for up to 16 weeks. Panels a-i represent time-course profiles for NADP+, NADPH, NAD+, NADH, NAD(H), NADP(H), NAD(P)(H), NADPH/NADP+ and NADH/NAD+, respectively. Storage conditions: 0 °C, 2.5 kPa O_2_ and 5 kPa (●) or 0.03 kPa (○) CO_2_. All the data represent the mean of four treatment replicates, each being the average of three to four subsamples. The error bar below 0 week represents the least significant difference at the *P* ≤ 0.05 level. Note that the *y*-axis varies among the panels. NAD^+^/NADH oxidized/reduced nicotinamide dinucleotide, NADP^+^/NADPH oxidized/reduced nicotinamide dinucleotide phosphate, NAD(H) total oxidized/reduced nicotinamide dinucleotide, NADP(H) total oxidized/reduced nicotinamide dinucleotide phosphate, NAD(P)(H) total oxidized/reduced nicotinamide dinucleotide (phosphate)

The major non-phosphorylated and phosphorylated pyridine dinucleotides in both freshly harvested and stored ‘Empire’ fruit were the reduced forms. The concentrations of NAD^+^, NADH and NADP^+^ in both control and CO_2_-treated fruit generally decreased with storage time, whereas NADPH increased, resulting in lower levels of NAD(H) and higher levels of NADP(H) (Fig. 1a–f). Overall, there was an approximately 60% decrease in the concentrations of total pyridine dinucleotides (Fig. 1g), a 50% decrease in the NADH/NAD^+^ ratio (Fig. 1i), and a two-fold increase in NADPH/NADP^+^ ratio (Fig. 1h). The NADH/NAD^+^ and NADPH/NADP^+^ ratios ranged from 20–70 and 60–150, respectively.

### Levels of GABA and closely related metabolites and gene transcripts

**Fig. 2:**
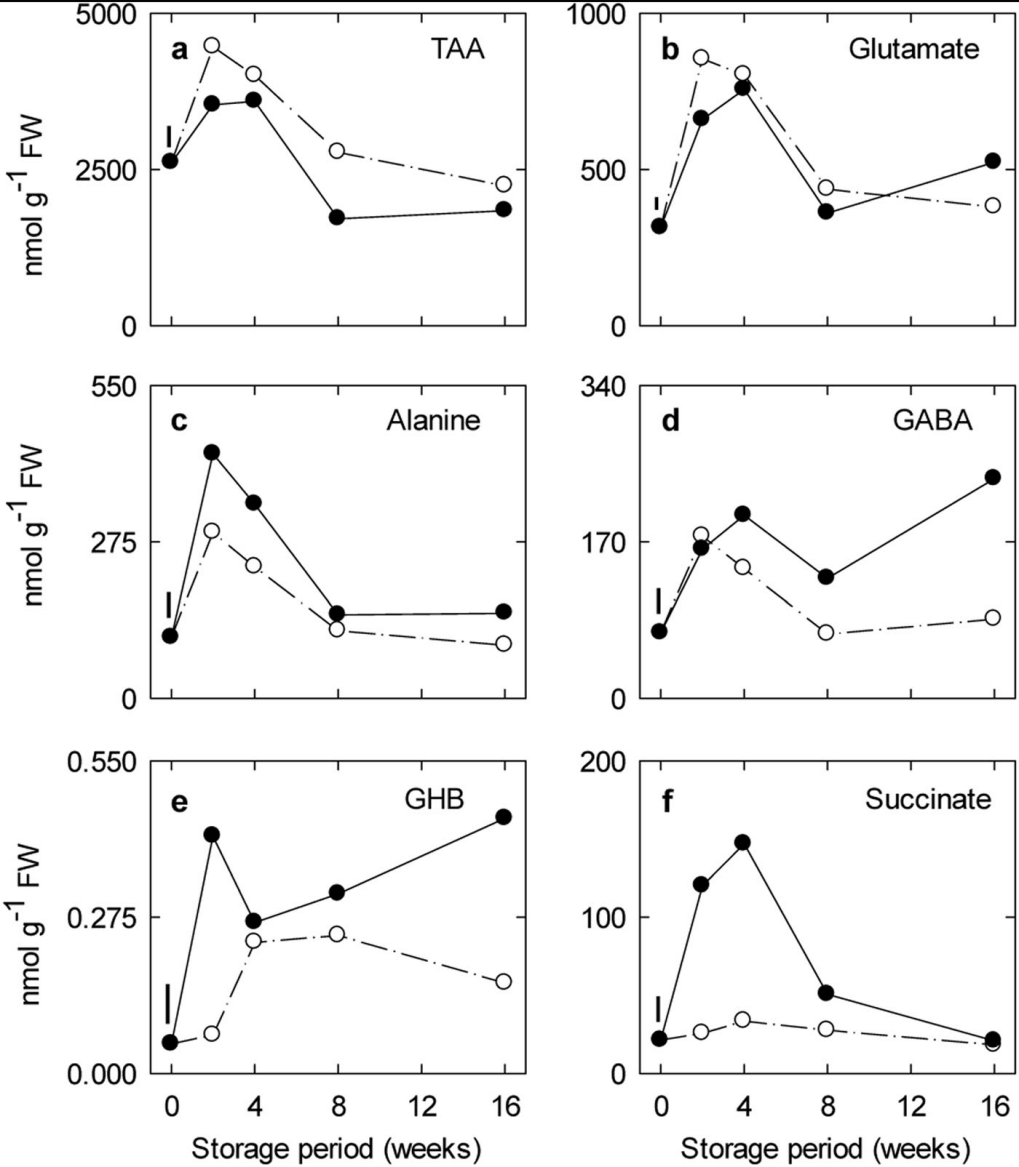
Impact of elevated CO_2_ on the levels of GABA and related metabolites in ‘Empire’ apple fruit under low-temperature/low-O_2_ storage for up to 16 weeks. Panels a-f represent time-course profiles for TAA, glutamate, alanine, GHB and succinate, respectively. Storage conditions: 0 °C, 2.5 kPa O_2_ and 5 kPa (●) or 0.03 kPa (○) CO_2_. All the data represent the mean of four treatment replicates, each being the average of three to four subsamples. The error bar above 0 wk represents the least significant difference at the *P* ≤ 0.05 level. Note that the *y*-axis varies among the panels. GABA 4-aminobutyrate, GHB 4-hydroxybutyrate, TAA total amino acids

The major amino acids in both freshly harvested and stored fruit were aspartate, asparagine and glutamate (Supplementary Information Table S4). In control fruit, the concentrations of total amino acids (TAA), GABA and GABA-related amino acids (i.e., glutamate and alanine) increased to a maximum within 2–4 weeks, and then declined to their original levels after 8–16 weeks (Fig. 2a–d)). There was also a transient peak in GHB, but this was delayed in comparison to the amino acids (Fig. 2e). The concentration of succinate was low and steady over the entire storage period (Fig. 2f). Storage with 5 kPa CO_2_ significantly increased the concentrations of alanine, GHB and succinate early during storage, and GHB and GABA later in the storage period. Notably, the concentration of GHB is much lower than succinate and also increasing with high CO_2_ when succinate is decreasing.

**Fig. 3:**
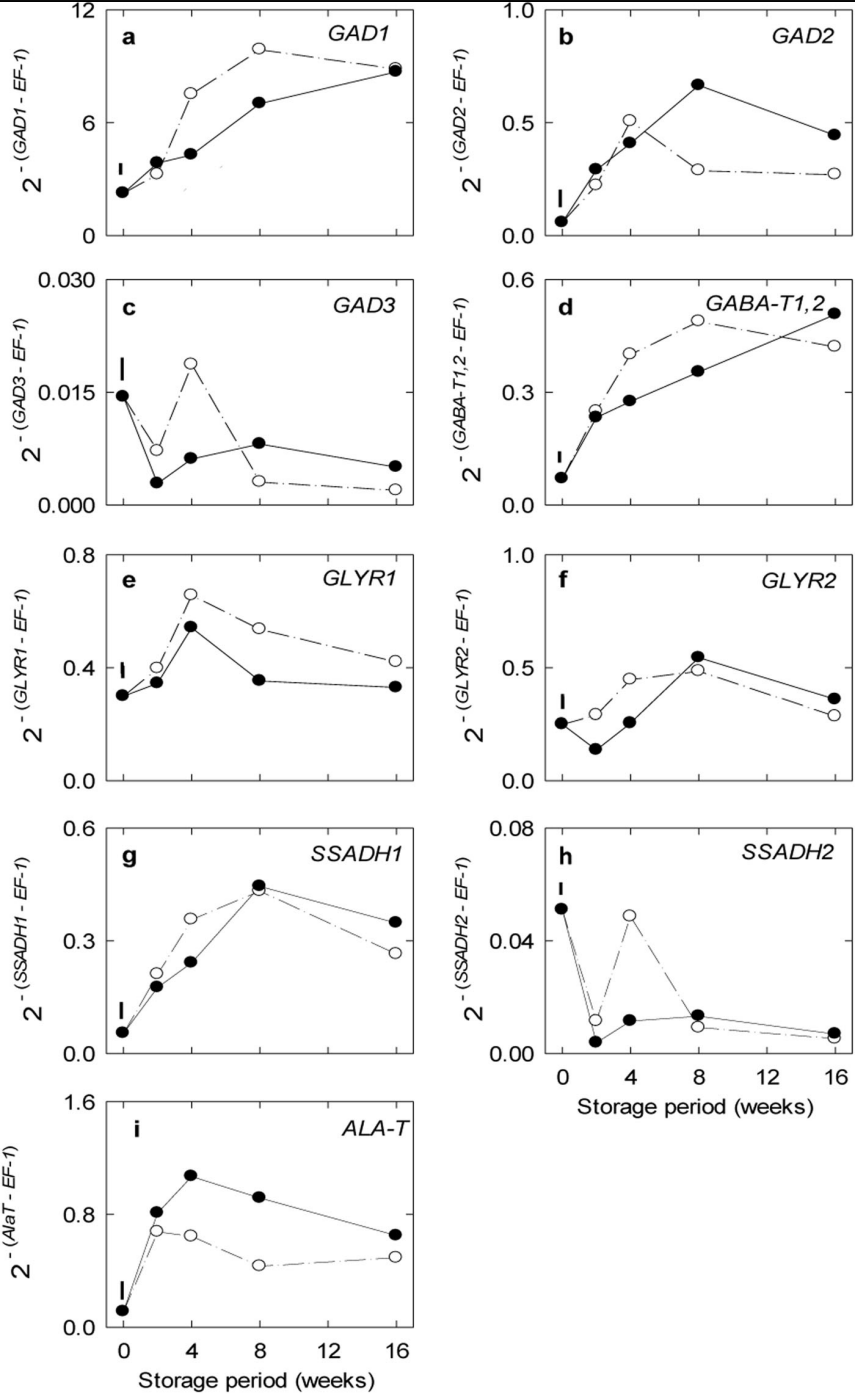
Impact of elevated CO_2_ on the expression of genes associated with the biosynthesis and catabolism of GABA from glutamate in ‘Empire’ apple frui under low-temperature/low-O_2_ storage for up to 16 weeks. Panels a-i represent time-course profiles for *GAD1*, *GAD2*, *GAD3*, *GABA-T1,2*, *GLYR1*, *GLYR2*, SSADH1, SSADH2 and ALA-T, respectively. Storage conditions: 0 °C, 2.5 kPa O_2_ and 5 kPa (●) or 0.03 kPa (○) CO_2_. All the data represent the mean of three treatment replicates, each being the average of three subsamples. The error bar above 0 weeks represents the least significant difference at the *P* ≤ 0.05 level. Note that the *y*-axis varies among the panels. *ALA-T* alanine dehydrogenase, *GABA-T* GABA transaminase, *GAD* glutamate decarboxylase, *GLYR* glyoxylate reductase, *SSADH* succinic semialdehyde dehydrogenase

The transcript abundance of *ALA-T*, which could be considered as a reliable indicator of hypoxic conditions^31^, reached a maximum in the control within 2 wk of storage, and then levelled off for the remaining storage time (Fig. 3i). Treatment with CO_2_ further enhanced *ALA-T* transcript abundance at 4–8 weeks. Of the *GAD* transcripts in the control, *GAD1* was most abundant, *GAD2* was moderately abundant, and *GAD3* was lowly abundant (Fig. 3a–c). The abundance of *GAD1* and *GAD2* transcripts increased linearly up to 4–8 weeks and then levelled off, whereas the abundance of *GAD3* transcript declined after a transient peak. Treatment with CO_2_ delayed the increase in *GAD1* transcript abundance, and increased the maximal abundance of *GAD2* transcript late in the storage period. *GABA-T1,2* (Fig. 3d) and *SSADH1* (Fig. 3g) transcripts were moderately abundant in the control and displayed similar CO_2_ responses and patterns as *GAD1*, whereas the *SSADH 2* (Fig. 3h) transcript was much less abundant and rapidly declined in a CO_2_-independent manner with storage time. The transcripts for *GLYR1* and *GLYR2* were moderately abundant and transiently increased by ~1-fold early in the storage period (Fig. 3e–f). Treatment with CO_2_ decreased abundance of the *GLYR1* and *GLYR2* transcripts during mid and early storage, respectively.

### In silico analysis of putative apple PAOs

In silico analysis revealed that the six putative apple *PAO* genes encode proteins ranging from 488 to 533 amino acids and from 24% (PAO1 and PAO6) to 91% (PAO3 and PAO4) sequence identity (Supplementary Information Table S5). Apple PAO1 is 72% identical to Arabidopsis PAO1, apple PAO2 is 79% identical to Arabidopsis PAO2, and apple PAO3 is 60% identical to Arabidopsis PAO3. In particular, apple PAO5 and apple PAO6 have a high degree of identity (90%) to each other, as well as Arabidopsis PAO5.

Sequence comparison and phylogenetic analysis of the six putative apple *PAO* genes to known *Arabidopsis* PAOs reveal that they can be divided into three distinct groups as described for *Arabidopsis*^32^. Apple PAO2, PAO3 and PAO4, together with Arabidopsis PAO2, PAO3 and PAO4 form a cluster possessing a peroxisome targeting signal 1 (Supplementary Information Figure S1a–b). *Arabidopsis* members of this group are localized in the peroxisome^4^, and similar subcellular localization is predicted for apple members (PAO2-4) of this group. Moreover, apple PAO1 clusters with apple PAO1, whereas apple PAO5, apple PAO6 and Arabidopsis POA5 cluster separately. Arabidopsis PAO1 and PAO5 appear to encode cytosolic proteins^4^; therefore, apple PAO5 and PAO6 are predicted to be cytosolic.

### Levels of polyamines and expression of genes associated with their catabolism to GABA

**Fig. 4:**
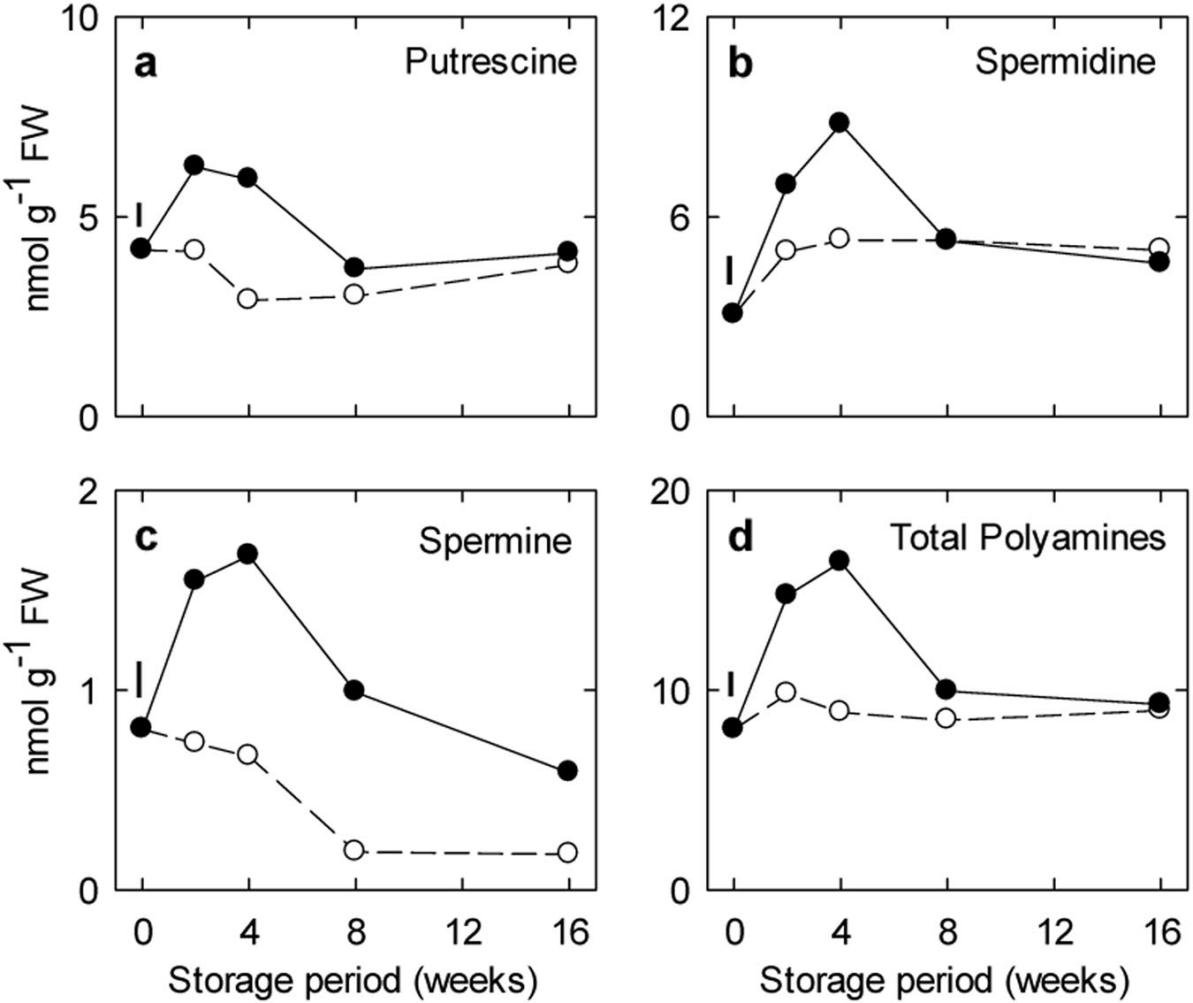
Impact of elevated CO_2_ on the polyamine levels in ‘Empire’ apple fruit under low-temperature/low-O_2_ storage for up to 16 weeks. Panels a-d represent time-course profiles for putrescine, spermidine, spermine and total polyamines, respectively. Storage conditions: 0 °C, 2.5 kPa O_2_ and 5 kPa (●) or 0.03 kPa (○) CO_2_. All the data represent the mean of four treatment replicates, each being the average of three to four subsamples. The error bar above 0 wk represents the least significant difference at the *P* ≤ 0.05 level. Note that the *y*-axis varies among the panels

The major polyamines in both freshly harvested and stored fruit were putrescine and spermidine, with minor concentrations of spermine. The concentrations of putrescine and spermidine were relatively steady in the control over the storage period, whereas spermine slowly declined (Fig. 4a–d). Treatment with CO_2_ increased the concentrations of all polyamines early in the storage period, but only spermine was higher than the control over the entire period. In general, concentrations of the polyamines were much lower than those for the GABA-related amino acids (Fig. 2b–d)

**Fig. 5:**
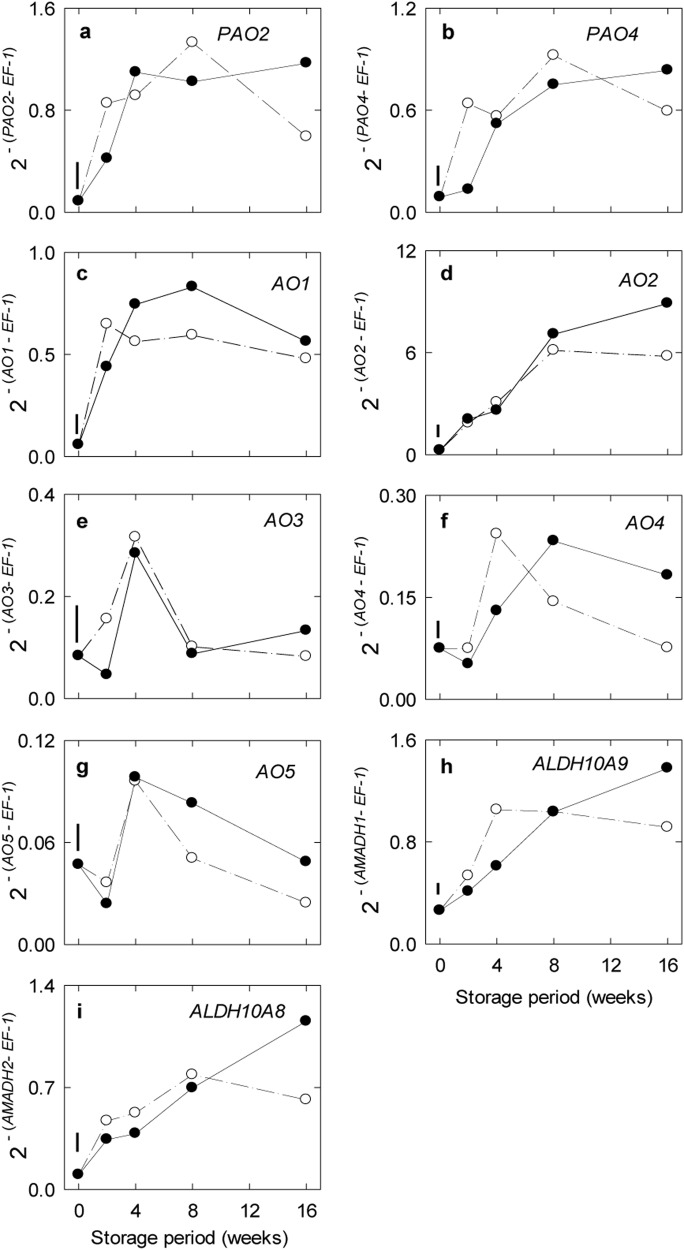
Impact of elevated CO_2_ on the expression of genes associated with the catabolism of polyamines to GABA in ‘Empire’ apple fruit under low-temperature/low-O_2_ storage for up to 16 wk. Panels a-i represent time-course profiles for *PAO2*, *PAO4*, *AO1*, *AO2*, *AO3*, *AO4*, *AO5*, ALDH10A9 and ALDH10A8, respectively. Storage conditions: 0 °C, 2.5 kPa O_2_ and 5 kPa (●) or 0.03 kPa (○) CO_2_. All the data represent the mean of three treatment replicates, each being the average of three subsamples. The error bar above 0 wk represents the least significant difference at the *P* ≤ 0.05 level. Note that the *y*-axis varies among the panels. *AO* Amine Oxidase, *ALDH* aldehyde dehydrogenase, *PAO* polyamine oxidase

Of the six apple *PAO* genes identified, only the transcripts for *PAO2* and *PAO4* genes were readily detected in fruit (Fig. 5a, b). The *PAO2* transcript was slightly more abundant than the *PAO4* transcript, although both peaked midway through the storage period. The *AO2* transcript was the most abundant of the five apple *AO* genes, and peaked late in storage (Fig. 5c–e). The *AO1* transcript was moderately abundant and peaked within 2–4 weeks. Any significant responses of these aforementioned *PAO* and *AO* transcripts to elevated CO_2_ seemed to be transient. The *AO3-5* transcripts were present in low abundance and peaked transiently at 4–8 weeks; *AO4* appeared to show CO_2_-dependent stimulation during late storage (Fig. 5e–g). The *ALDH10A* transcripts were moderately abundant and peaked by 2–4 weeks, although a positive response to elevated CO_2_ tended to be delayed (Fig. 5h–i).

### Correlation and principal component analyses of metabolite and gene transcript levels

**Fig. 6:**
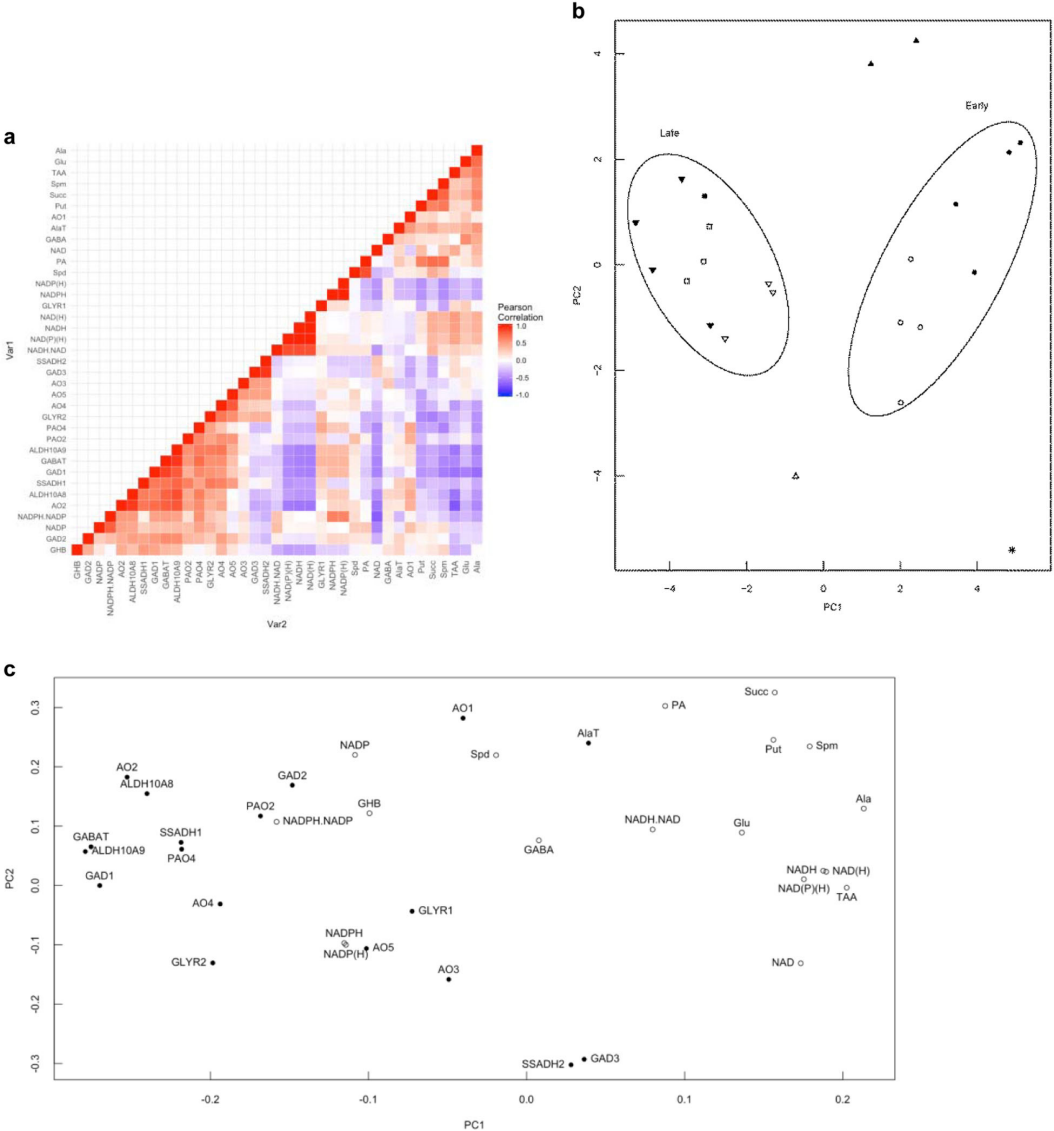
Relationships among GABA pathway metabolites and transcripts in ‘Empire’ apple fruit under low-temperature/low-O_2_ storage at two CO_2_ levels for up to 16 wk. **a** Pearson’s correlation analysis. A total of 666 pairs were analysed, from which 68 resulted in significant correlations (*P* ≤ 0.05). Of these, 58 were positive and 10 negative. Each square represents the correlation between each metabolite or transcript in the column and the metabolite or transcript in the corresponding row with a color scale (color scale key at the side of the figure). **b** Principal component analysis. Score plot: fresh harvest control (*) and all variables at 2 (circles), 4 (triangles), 8 (squares) and 16 (inverted triangles) weeks of storage; 0.03 kPa CO_2_ (open symbols) and 5 kPa (filled symbols) CO_2_. The 2-week (early) and 8/16-week (late) harvests are enclosed separately by solid lines. **c** Principal component analysis. Loading plot of all variables: metabolites (open circles); transcripts (closed circles). The two principal components (PCs) explained 43.4% of the overall variance (30.2 and 13.2% for PC1 and PC2, respectively). The further from the origin, the more variables would be influenced by low temperature/low O_2_ (PC1) or elevated CO_2_ (PC2); variables with a higher loading value for PC1, but lower for PC2 would be more influenced by low temperature/low O_2_, whereas variables with a higher loading value for PC2 but a lower loading value for PC1 would be more influenced by elevated CO_2_. Ala alanine, *ALA-T* alanine transaminase, *ALDH* aldehyde dehydrogenase, *AO*, amine oxidase, GABA 4-aminobutyrate, *GABA-T* GABA transaminase, *GAD* glutamate decarboxylase, GHB 4-hydroxybutyrate, Glu glutamate, *GLYR* glyoxylate reductase, *PAO* polyamine oxidase, NAD^+^/NADH oxidized/reduced nicotinamide dinucleotide, NADP^+^/NADPH oxidized/reduced nicotinamide dinucleotide phosphate, NAD(H) total oxidized/reduced nicotinamide dinucleotide, NADP(H) total oxidized/reduced nicotinamide dinucleotide phosphate, NAD(P)(H) total oxidized/reduced nicotinamide dinucleotide (phosphate), PA total polyamines, Put putrescine, Spd spermidine, Spm spermine, *SSADH* succinic semialdehyde dehydrogense, Succ succinate; TAA total amino acids

Correlation analysis was performed on the entire data set of metabolites and gene transcripts from apples subjected to low-temperature/low-O_2_ storage at two CO_2_ levels by calculation of the Pearson’s correlation coefficient for each metabolite/metabolite or metabolite/transcript pair (Fig. 6a). Notably, GABA and GHB were not significantly correlated with each other, nor with any of the other metabolites measured, including glutamate and NADPH/NADP^+^. Positive correlations were found among various metabolites and transcripts: glutamate with alanine; alanine with glutamate, TAA, succinate and *GAD1*; succinate with alanine, putrescine, spermine and total polyamines; total polyamines with succinate, putrescine, spermidine and spermine; putrescine with succinate, spermine and total polyamines; spermidine with total polyamines; spermine with succinate, putrescine and total polyamines; and, spermine with putrescine, spermidine and total polyamines. NADPH/NADP^+^ was positively correlated with NADPH, NADP^+^ and NADP(H), and negatively correlated with NAD^+^. Also, there were significant positive correlations among various transcripts: *GAD1* with *GABA-T*, *SSADH1, AO2*, *ALDH10A8* and *ALDH10A9; GABA-T* with *GAD1*, *SSADH1*, *AO2* and *ALDH10A8, ALDH10A9* and *PAO4; GLYR2* with *SSADH1, AO4* and *ALDH10A9*; *SSADH1* with *GAD1*, *GABA-T*, *GLYR2*, *AO2*, *AO4*, *ALDH10A9*, *ALDH10A8* and *PAO2*; *AO2* with *GAD1*, *GABA-T*, *SSADH1, ALDH10A8* and *ALDH10A9*; *AO4* with *SSADH1*, *AO5, GLYR2* and *ALDH10A9*; *ALDH10A9* with *GAD1*, *GABA-T*, *GLYR2*, *SSADH1*, *AO2* and *AO4, ALDHA8* and *PAO4*; *ALDH10A8* with *GAD1*, *GABA-T*, *SSADH1*, *AO2*, *ALDH10A9* and *PAO4*; *PAO2* with *SSADH1*; *PAO4* with *GABA-T*, *ALDH10A9* and *ALDH10A8*; and, *SSADH2* with *GAD3*.

The metabolite and transcript data set was also examined by PCA. The score plot suggests that PC1 captures the variability between early and late storage periods, and PC2 captures the variability between CO_2_ treatments (Fig. 6b). Hence, the biological variables in the loading plot can be discussed in these terms (Fig. 6c). GABA responded slightly to CO_2_, but not storage, whereas GHB responded slightly and moderately to storage and CO_2_, respectively. The pyridine dinucleotides and the NAD(P)H/NAD(P)^+^ ratio moderately responded to storage, and NADP^+^ in particular strongly responded to CO_2_. Several metabolites (i.e., succinate, spermine, putrescine, alanine and glutamate), including TAA, moderately to strongly responded to storage, and succinate, putrescine and spermine strongly responded to CO_2_. Many gene transcripts (i.e., *GAD2*, *PAO2*, *AO4*, *GLYR2*, *PAO4*, *SSADH1*, *ALDH10A8*, *AO2*, *GAD1*, *GABA-T* and *ALDH10A9*) moderately to strongly responded to storage, and several of these (i.e., *GAD2*, *PAO2*, *ALDH10A8*, *AO2*) moderately responded to CO_2_. Other transcripts strongly responded to CO_2_, but only slightly to storage (i.e., *ALA-T*, *AO1*, *SSADH2* and *GAD3*).

## Discussion

### Metabolite relationships

The ratios of reduced to oxidized pyridine dinucleotides in freshly harvested and low-temperature CA-stored ‘Empire’ apples (Fig. 1) were much higher than in photosynthesizing unstressed *Arabidopsis* leaves^13,33^, and *ALA-T* expression was induced and maintained during storage (Fig. 3). Furthermore, the NADH/NAD^+^ ratio declined over the storage period, and the NADPH/NADP^+^ ratio increased, particularly during late storage, regardless of the treatment regimen (Fig. 1). These findings are consistent with the idea that intact ‘Empire’ apples are already in a reduced state and metabolism is O_2_-limited (i.e., hypoxic) at harvest, and that this becomes increasingly so with storage^1,16,17,19,20,31^.

The major amino acids in both freshly harvested and stored fruit were aspartate, asparagine and glutamate (Fig. 2; Supporting Information Table S4), whereas polyamines consisted of similar levels of putrescine and spermidine, together with minor levels of spermine (Fig. 4). TAA and GABA-related amino acids transiently accumulated early in storage, regardless of the CO_2_ regimen (Fig. 2). This was accompanied by relatively stable levels of polyamines, succinate and GHB with ambient CO_2_, but transient accumulation of these same metabolites with elevated CO_2_ (Figs. 2 and 4). Notably, the succinate level further declined thereafter, whereas GABA and GHB increased over the longer term. These findings are in general agreement with previous reports of dynamic changes in proteins, proteolytic activity, amino acids and polyamines in apple fruit stored under low-temperature conditions, in the absence or presence of low O_2_ and elevated CO_2_^34–37^. Thus, it can be suggested that the low temperature stimulated protein hydrolysis during early storage, thereby temporarily increasing the pools of amino acids available for various metabolic processes. The elevated glutamate level in particular seemed to influence the relative level of GABA much more than polyamines, despite lower concentrations of the polyamines. Further research is required to establish whether this result can be explained by differences in the utilization (e.g., substrate affinity and/or localization) of glutamate by GAD or biosynthetic enzymes for arginine, the primary precursor for polyamines^38,39^. Also, it can be suggested that elevated CO_2_ had distinct effects on the production of polyamines, as well as succinate and GHB, during early storage, and on GABA and GHB production over the longer term (see “Metabolite-transcript relationships”). Overall, a complex pattern of GABA-related metabolites could be recognized in ‘Empire’ apple exposed to a combination of low temperature/low O_2_ and elevated CO_2_.

### Transcript relationships

**Fig. 7:**
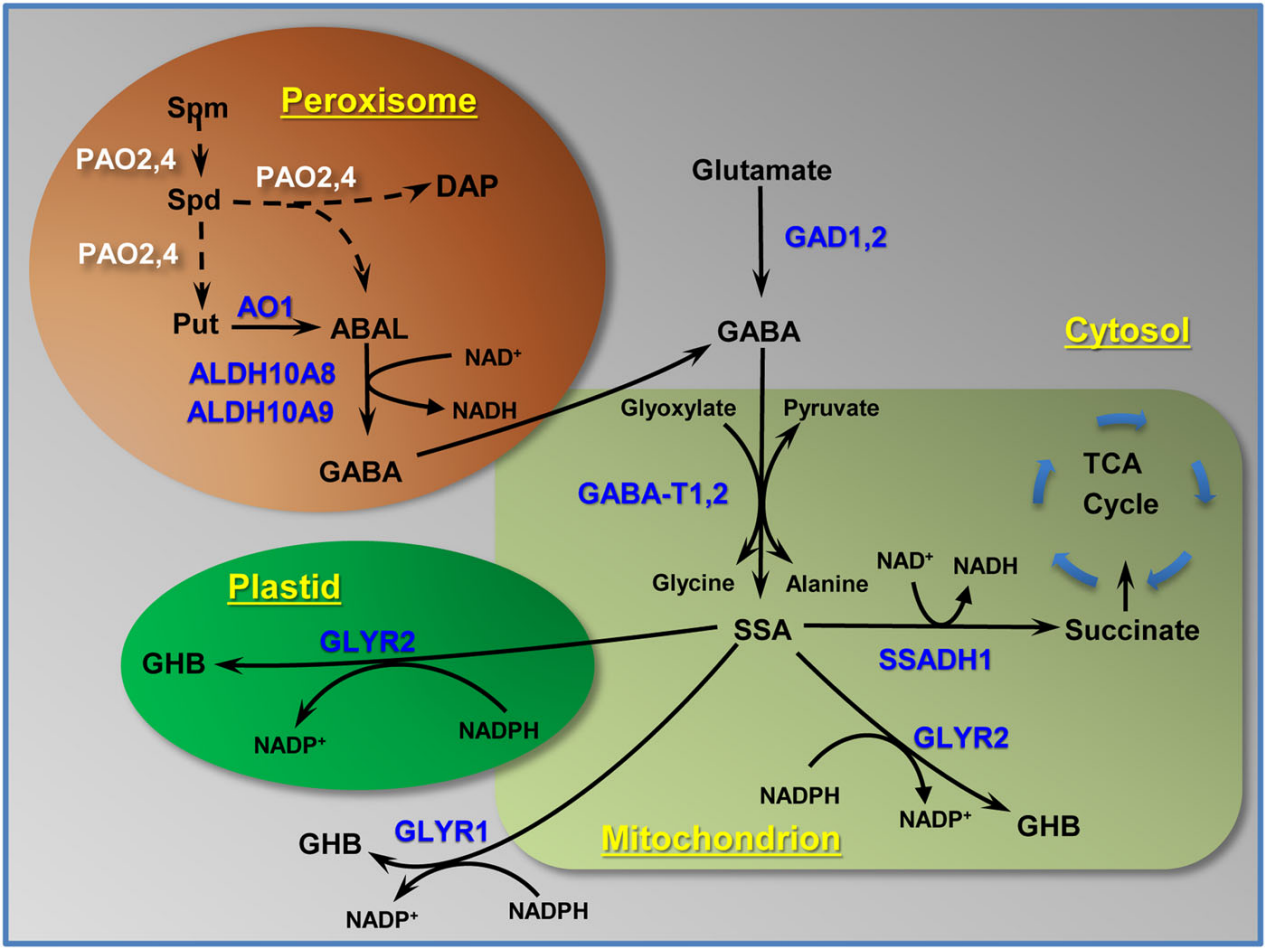
Model for the induction and subcellular localization of enzymes associated with GABA and GHB metabolism in ‘Empire’ apple fruit stored under low temperature, low O_2_ and elevated CO_2_ conditions. Bold blue lettering represents known biochemically characterized enzymes, whereas bold white lettering and dashed arrows represent putative polyamine oxidases, which were localized to the peroxisome using *in silico* comparisons with Arabidopsis orthologs (see Supplementary Information Fig. S1). 4-ABAL 4-aminobutanal, DAP 1,3-diaminopropane, SSA succinic semialdehyde, TCA tricarboxylic acid. For other abbreviations, see Fig. 6

Figure 7 contextualizes our current understanding of stress-induced GABA production in apple fruit from the decarboxylation of glutamate and the catabolism of polyamines. The route from glutamate to GABA is probably catalyzed by two of the three cytosolic *Md*GADs (*Md*GAD1 and *Md*GAD2), which are abundant and interact with Ca^2+^-calmodulin^24^. Putrescine and spermidine also represent potential sources of GABA via the metabolite 4-aminobutanal. Spermidine and spermine, respectively, are known to be back-converted to putrescine and/or spermidine in dicotyledonous plants^1,4^, and preliminary assessment here, based on *in silico* analysis, suggests that specific apple FAD-polyamine oxidases (PAO2,4) are peroxisomal (Supplementary Information Figure S1). Five copper AOs are present in ‘Empire’ apple fruit, but only one of the two most abundant forms (i.e., AO1) is peroxisomal and prefers diamines as substrates^5^. Two putative peroxisomally-located, NAD^+^-dependent *Md*ALDH10As can convert 4-aminobutanal to GABA^7^. Therefore, AO1 and the ALDH10As represent a likely path for putrescine oxidation to GABA in apple fruit.

In the present study, the apple fruit genes associated with GABA anabolism from both glutamate and polyamines, and with GABA catabolism to succinate were all co-ordinately upregulated by low temperature/low O_2_ with ambient CO_2_ (Figs 3, 5, 6, 7). Furthermore, there was evidence for CO_2_-upregulated expression of *GAD2*, *AO1*, *ALDH10A8* and *PAO2* with mid- to long-term storage (Fig. 6). Previous studies have attempted to directly link transcriptome changes to stress-induced increases in GABA levels in plants^1,4,40,41^, For example, a limited number of key GABA pathway genes in *Arabidopsis* (i.e., *GAD4*, *SSADH* and *GABP*) and tomato fruit (i.e., *GAD2,3*, but not *GAD1*, *GABA-T*, *SSADH* or *GLYR1,2*) appear to be upregulated in a co-ordinated manner by cold and elevated CO_2_, respectively^11,42^. Thus, expression of genes linked to stress-induced GABA metabolism in apple fruit displays some unique regulatory properties and close examination of the corresponding promoter regions could reveal common motifs and elements associated with low temperature, low O_2_ or elevated CO_2_.

### Metabolite-transcript relationships

Although transcriptional mechanisms are essential for GABA metabolism to proceed in apple fruit during storage, the increasing abundance of key gene transcripts is probably not the entire explanation for the CO_2_-stimulated GABA accumulation during late storage (Figs. 2, 3, 4, 5, 6). The accumulation of GABA could also involve cytosolic acidification-mediated or Ca^2+^/calmodulin-activated increases in the activities of GAD1 and/or GAD2^1,24,43,44^. The dramatic loss in polyamine levels over the same period might be linked with the induction of *AO1*, but given that the activity of the corresponding enzyme relies on molecular O_2_ for catalysis^5,45,46^, ALDH10As are NAD^+^-dependent^7^, and CA-stored apple fruit are probably O_2_ limited^16,17^ with an elevated NAD(P)H/NAD(P)^+^ ratio (see “Metabolite relationships”), it seems more likely that AO1, PAO2, and ALDH10A8 activities would be restricted in apple fruit and cause the accumulation of the polyamines^1^. Interestingly, both glutamate and alanine pools declined during this period, but they were not markedly affected by the CO_2_ regimen, providing support for the maintenance in plants of glutamate within narrow concentration limits^47^. Previous research has shown that two GABA peaks are found in rice being germinated under anoxia and the first peak is associated with slight upregulation of at least one of five *GAD*s and downregulation of both *GABA-T* and *SSADH*, whereas a single transient GABA peak is found in chilled *Arabidopsis* shoots and it is associated with upregulation of both *GAD4* and *SSADH*^11^.

The initial CO_2_-regulated accumulation of succinate and GHB in apple fruit could not be attributed to elevated expression of *GAD1*, *GAD2*, *SSADH* and *GLYR*. Notably, the initial accumulation of GHB preceded that of GABA, and the second CO_2_-regulated accumulation of GHB was inversely related to succinate accumulation (Figs. 2, 3 and 6). The internal elevated CO_2_/O_2_ environment, rather than O_2_ alone, probably accounts for the changing redox balance evident in the low-temperature, CA-stored apple fruit (Fig. 1)^48,49^. These reducing conditions could differentially restrict the activities of TCA cycle enzymes, as well as SSADH, and enhance the activities of GLYR over the storage period, thereby modifying the accumulation of succinate and GHB (Figs. 1 and 6)^1^. Unfortunately, metabolite pool sizes are not by themselves very informative in addressing mechanisms^44^. For example, the first peak of GHB is difficult to reconcile on the basis of redox balance alone. Previous studies have reported that succinate accumulates continuously for up to 50 h in rice germinating under anoxia, with GHB accumulating prior to GABA^11^, and a lack of correlation between *GLYR* expression and GHB accumulation in submerged *Arabidiopsis*^18,19^. Another study has demonstrated that a transient increase in GHB level in shoots of chilled *Arabidopsis* plants follows a transient increase in GABA and it is independent of *GLYR* expression^11^. However, there is a concomitant and sustained accumulation of succinate, which can be interpreted as support for the operation of a non-conventional TCA cycle^1^. Additional research is required to determine if stress-induced peroxidation of phospholipids containing 4-hydroxybutyryl chains generates GHB in apple fruit, as it is does in mammals^49–53^, or if the prolonged storage period alters the carbon/nitrogen balance, resulting in carbon limitation of the TCA cycle and diversion of GABA carbon from succinate to GHB^1,20,54^.

Together, these findings suggest that both transcriptional and biochemical mechanisms are associated with GABA and GHB metabolism in apple fruit stored under multiple abiotic stress conditions. Exploration of the function of GHB in model plants such as *Arabidopsis* during exposure to elevated CO_2_, low O_2_ and/or low temperature, using single and double overexpression or knockout mutants of *GABA-T* and *GLYR*, is warranted.

### Disclaimer

AgroFresh Inc. and the Ontario Apple Growers had no involvement in collection of the data or the decision to publish.

## Electronic supplementary material

Supplementary Materials
