# Supplementary material for: Targeted quantitative profiling of metabolites and gene transcripts associated with 4-aminobutyrate (GABA) in apple fruit stored under multiple abiotic stresses
Source: Hortic Res. 2018 Dec 1;5:61. doi: 10.1038/s41438-018-0069-3 (PMC6269452; doi:10.1038/s41438-018-0069-3)
Supplement: Supplementary file 1 — Supplementary Materials [file 41438_2018_69_MOESM1_ESM.pdf]

SUPPLEMENTARY INFORMATION

Targeted quantitative profiling of metabolites and gene transcripts associated with 4-aminobutyrate (GABA) in apple fruit stored under multiple abiotic stresses

Carolyne J. Brikis[1,4], Adel Zarei[1,4], Greta Z. Chiu[1], Kristen L. Deyman[1], Jingyun Liu[1], Christopher P. Trobacher[1], Gordon J. Hoover[1], Sanjeena Subedi[2], Jennifer R. DeEll[3], Gale G. Bozzo[1] and Barry J. Shelp[1]

[1]Department of Plant Agriculture, University of Guelph, Guelph, Ontario, Canada N1G 2W1; [2] Department of Mathematical Sciences, Binghamton University, Binghamton, NY, USA 13902; and [3]Ontario Ministry of Agriculture, Food and Rural Affairs, Box 587, 1283 Blueline Rd. at Highway 3, Simcoe, Ontario, Canada N3Y 4N5

[4]These authors contributed equally to the work.

Supplementary Materials and Methods S1- GHB, Succinate and Pyridine Dinucleotide Analyses

To determine GHB in each treatment replicate, 37.5 mg of frozen apple powder for each of the four subsamples for a treatment replicate was pooled. The pooled tissue was spiked with 148 pmol of the internal standard GHB-d6 (C/D/N Isotopes), as well as 155 pmol GHB (Sigma) to improve the sensitivity of the method, and combined with 500 μL of 80 % (v/v) ethanol and homogenized with a small pestle for 5 min. The homogenate was clarified by centrifugation at 21,000 *x g* for 20 min and the pellet was discarded. The supernatant was combined with 200 μL of chloroform, vortexed for 1 min, followed by addition of 300 μL of Milli-Q water and an additional 2 min of vortexing. The mixture was centrifuged as described above, and the polar layer was collected and passed through a 0.45-μm nylon syringe filter. A 120-μL aliquot of the filtered polar layer was aliquoted into glass inserts and dried down by rotary concentrator (Savant SPD 2010, Thermo Scientific, Asheville, NC, U.S.A.) for 15 h. The dried residue was resuspended in 40 μL of 20 g L^{-1} methoxyamine hydrochloride in pyridine by vortexing for 10 s, and then incubated for 90 min at 37 ºC. Subsequently, 80 μL N-Methyl-N-(trimethylsilyl) trifluoroacetamide was added, and the sample was incubated for 45 min at 37 ºC. The solution was allowed to stand overnight at room temperature, and then a 1-μL aliquot of the derivatized plant extract was split-injected onto the gas chromatograph-mass spectrometer/mass spectrometer (Scion 436-GC, Bruker Daltonics Inc., Fremont, CA, U.S.A.) through a 30 m, 0.25 mm ID, 0.25 mm df column (BR-5ms, Bruker) carried by helium gas. Initially, the column oven temperature was held at 50 ºC for 5 min, followed by an increase of 5 ºC min^{-1} up to 140 ºC, and then a 20 ºC min^{-1} increase to 300 ºC, which was held for 1 min. Three solvent washes of the injector syringe were performed prior to each sample injection. The transfer line was kept at 250

ºC, and the MS-MS source was maintained at 200 ºC. GHB-d6 was monitored by precursor and product ions of m/z 239 and m/z 146, respectively, while GHB was monitored by precursor and product ions of m/z 233 and m/z 146, respectively. Stock solutions (1 g L^{-1}) of GHB (Sigma) and GHB-d6 (C/D/N Isotopes) were each generated by dissolving the appropriate compound in HPLC-grade water. A 25-µL aliquot of the stock solution was mixed with 975 µL of HPLC-grade water, followed by a number of 500 µL serial dilutions in HPLC-grade water to produce a set of GHB solutions in the µg L^{-1} range (~6 to 195 pg μL^{-1} range used here). Thereafter, 50 µL of each GHB solution was pipetted into glass inserts (Fisher) accompanied by 15 µL of a 195 pg μL^{-1} GHB-d6 solution. To account for matrix effects, 120 µL of apple extracts was added to each glass insert. These were dried down, derivatized, and injected onto the GC-MS-MS alongside apple samples. To create the calibration curve, the ratio of the peak area of GHB to the peak area of GHB-d6 was plotted against the amount of GHB injected for each standard. To determine the amount of GHB present in the tissue, the ratio of the peak area of GHB to the peak area of GHB-d6 was determined and substituted in the equation of the calibration curve to calculate the amount of GHB injected. For apple samples, 0.19 pmol of GHB was subtracted from this value to account for the exogenous GHB added during extraction. One standard and one blank were run every five to six samples. Use of an isotopic internal standard allows for corrections stemming from extraction efficiency, degree of derivatization, and any small changes in chromatography or detection due to its structural resemblance and small difference in mass[1].

Succinate was extracted from each treatment replicate by homogenizing 100 mg of apple tissue, comprised of 25 mg from each of the replicate subsamples, in 500 µL of ice cold 1 M perchloric acid. The extract was adjusted to pH 8 with 4 M KOH, and the final volume was made up to 1 mL. Then, 0.03 g mL^{-1} of polyvinylpyrrolidone (Sigma) was added and the solution

incubated for 20 min, shaking on ice. Samples were stored on ice for an additional 20 min to allow precipitation of $KClO_4$, then centrifuged at 15, 000 *x g* for 15 min at 4 ^{o}C. The clarified supernatant was used in an endpoint enzyme-linked cuvette assay (K-SUCC, Megazyme International Ireland, Wicklow, Ireland) that was adapted for a microplate reader (SpectraMax Plus384). A linear standard curve ranging from 0 to 115 nmol succinate was constructed using internal standards submitted to the extraction process in apple tissue background. In order of addition into a 96-well plate, each reaction included 100 µL of clarified extract or standard, 86 µL ddH_2O, 20 µL of solution one (buffer), 20 µL of solution two (NADH), 20 µL of solution three (ATP, phosphoenolpyruvate, coenzyme A), and 2 µL of suspension four (pyruvate kinase, L-lactate dehydrogenase). This mixture was mixed and the initial absorbance determined at 340 nm. The reaction was initiated by mixing in 2 µL of suspension five (succinyl-coA synthetase), giving a final volume of 250 µL. The final absorbance was determined 30 min after initiation. A blank obtained with a non-initiated assay was subtracted from the difference between initial and final absorbance, and the result was compared to the internal standard curve to quantify the amount of succinate.

Pyridine dinucleotides were extracted and assayed essentially as reported previously[2], with minor modifications. Samples consisted of 100 mg of frozen pulverized apple tissue comprised of 25 mg each from the four storage treatment replicates. $NAD(P)^+$ and NAD(P)H were extracted in 1 mL ice cold 0.2 N HCl or NaOH, respectively, containing 0.03 mg mL^{-1} polyvinylpyrrolidone, then shaken on ice for 20 min before being processed. Acidic and basic samples were neutralized with 1 M NaOH and 2 N HCl, respectively, to a final pH of 7, and the final extract volume was made up to 1 mL. $NAD(P)^+$ assays included 100 µL of extract and were initiated with a master mix containing 45 µL of 0.1 M 4-(2-hydroxyethyl)-1-

piperazineethanesulfonic acid + 2 mM ethylenediaminetetraacetic acid (pH 7.5), whereas NAD(P)H assays containing 10 µL of extract were initiated with a master mix containing 130 µL of 0.1 M 4-(2-hydroxyethyl)-1-piperazineethanesulfonic acid + 2 mM ethylenediaminetetraacetic acid (pH 7.5), as well as the other assay reagents. The linear rate was recorded as the decrease in A_{600} within the first minute. Pyridine dinucleotides were quantified with an internal standard curve, and values were corrected for the dilution factor, as well as the recovery of a spiked standard, and subtracted from a blank consisting of buffer in place of extract. This method was validated by measuring the concentrations of pyridine dinucleotides in photosynthesizing leaves from *Arabidopsis* plants grown under non-stress conditions[3]. These data are in close agreement with values reported by Queval and Noctor (2007)[2].

Supplementary Materials and Methods S2

Identification of apple genes

Putative apple*SSADH1* and *MdSSADH2* genes were identified using a BLAST search of the Arabidopsis *SSADH* sequence (GenBank Acc No. NM_106592) against the apple genome (www.rosaceae.org). Two sequences were highly similar to the *Arabidopsis* sequence: MDP0000147030 and MDP0000229588, designated as apple *SSADH1* and apple *SSADH2*, encode for 538 and 613 amino acid sequences, respectively. *Arabidopsis* and apple sequences are 87% identical (SSADH1) and 79% identical (SSADH2) and apple SSADH1 and *Md*SSADH2 are 92% identical.

The 543 amino acid sequence of *Arabidopsis* alanine aminotransferase 1 (*At*AlaAT1; GenBank Acc. No. AF275372) was used as a query for a BLAST search of GenBank for similar predicted peptides. Similar proteins including Arabidopsis AlaAT2, alanine aminotransferases from barley (GenBank Acc. No. CAA81231.1) and corn (GenBank Acc. No. AAC62456.1), and predicted peptides from grape (GenBank Acc. No.XP_002265294.1) and poplar (GenBank Acc. No. XP_002304255.1) were used to design degenerate primers CT-F27 and CT-R27 that amplified a 485 bp fragment (see Supplementary Information Table S1 for a list of primers). A translation of the fragment revealed that it was 79 to 89% identical to the sequences used to design the degenerate primers. The sequence was used to design primers for 5’ and 3’ for RACE. The primers CT-F30 and CT-F31 were used for 5′ RACE and nested PCR, respectively; CT-R30 and CT-R31 were used for 3′ RACE and nested PCR, respectively. Sequences obtained from the RACE reactions were combined to assemble a contig representing an apple ALANINE TRANSAMINASE (*ALA-T*). The translated apple ALA-T is 490 amino acids in length and 77 to 90% identical to the sequences used to design the degenerate primers. Apple ALA-T is 97%

identical to the last 507 amino acids of a 560 amino acid predicted peptide from the apple genome, MDP0000168683. The apple *ALA-T* sequence was used to design primers for quantitative reverse transcriptase polymerase chain reaction (qPCR).

The apple genome database (www.rosaceae.org) was searched utilizing known Arabidopsis PAOs (PAO1, At5g13700; PAO2, At2g43020; PAO3, *At*3g59050; PAO4, At1g65840; PAO5, At4g29720) sequences as queries at the nucleotide and amino acid levels. Six putative apple *PAO* genes were identified with following accession numbers: MDP0000261625; MDP0000321972; MDP00001888553; MDP0000702799; and, MDP0000941459. Single-stranded cDNA from 'Empire' apple was generated as described above. cDNA containing open reading frames of the apple *PAO* homologs were amplified using gene specific primers (see Supplementary Information Table S2 for a list of primers). All six putative apple *PAO* genes appeared to be expressed in leaves and were cloned into pCR2.1-TOPO (Invitrogen) vector using the manufacturer's protocol. At least three clones from independent PCR origin were sequenced for each *PAO* gene. The apple *PAO* gene sequences were designated as *PAO1*, *PAO2*, *PAO3*, *PAO4*, *PAO5* and *PAO6* and deposited in the GenBank (Acc. No. KT184496 –KT184501, respectively).

Supplementary Tables

Table S1. Synthetic oligonucleotides utilized for identification and cloning of apple *ALA-T*.

Primer	Sequence (5' to 3')
CT-F27	GCTTCCCCAGGTGTGCAYHTNATGATGC
CT-R27	CCTCTCTTTCCGCACTCTCCRTARTANCC
CT-F30	GCTGATAAGTTCAGAGAAGGATGGGATTCTTTGTCCC
CT-F31	CCTCAATACCCTTTGTACTCTGCGTCAATAGCCC
CT-R30	CCTGCTCGCCATATCCCATAGACCGGGATACC
CT-R31	CTGGTTGTCCTCGGCAAGAACCTGTCCTGTTGGG

Table S2. Synthetic oligonucleotides utilized for cloning apple *PAO*s.

Oligo name	Sequence (5' to 3')	Description
CT-F50	CTAGCCAACCGATATACACAGCGCCC	Forward primer for *Md*PAO1
CT-R50	CGGTCACCAATCATGGTGTTTTGCCTATAACTCC	Reverse primer for *Md*PAO1
CT-F51	CAATTCAATTCTTTTCGTTTCCCAATTTTCTTTTCAATTCC	Forward primer for *Md*PAO4
CT-R51	CCCATCTTTCATGTCTGAAGTATCGGAAAATGG	Reverse primer for *Md*PAO4
CT-F52	AATTCCAATACCAATTTCGATTTGAATCACC	Forward primer for *Md*PAO2
CT-R52	GGAGGATGGCGGCCGGG	Reverse primer for *Md*PAO2
CT-F53	CAATTMTTTTCAAATCCCCATTTTCTTGGAAATTCC	Forward primer for *Md*PAO3
CT-R53	GAAATGTAAATCCTATCATTCATTCATGTACCAGAATG	Reverse primer for *Md*PAO3
CT-F54	CCAAAAACCCATTACTTTAATTCCAGCTGAAAC	Forward primer for *Md*PAO5
CT-R54	GAAATAAATGTTCCAAAAATAGATAGAAATCCTACTAATAAT TG	Reverse primer for *Md*PAO5
CT-F55	CCAAAAGACCATTAATTTGGTTCCAGCTGAAAC	Forward primer for *Md*PAO6
CT-R55	GTACTTTAAAGAAAGAGAAGCAAAGATGTTCAACATG	Reverse primer for *Md*PAO6

Table S3. Synthetic oligonucleotides utilized for qRT-PCR in apple fruit.

Name	Sequence (5' to 3')
GAD1 forward	CAGCCAATGCGGAACATGTA
GAD1 reverse	CCGGCTGAAATCCTCCCTAA
GAD2 forward	AGTAGTTGATGCCGGCTGCTA
GAD2 reverse	CATACTCAGGAGCCCCTTTT
GAD3 forward	CGGTGGGACAGACACAGAGA
GAD3 reverse	CACTCCGACTAGTAGCATTTTGCA
GABA-T forward	GAGCATTGCCCCAAGATTTC
GABA-T reverse	TCCCCTATGATTGGACTGTCACA
GLYR1 forward	GGTCATACACATCGAACAGTACCTTT
GLYR1 reverse	TGATGATACAAATGAGTGCCTGTTT
GLYR2 forward	AAAATAGGACTGGACCCGAAAGT
GLYR2 reverse	TGGGCCTTTCATTGAGTACATTG
SSADH1 forward	CAGTGGCACCCCTTTTGC
SSADH1 reverse	GCAGCTAACCCTGCATTGGT
SSADH2 forward	TCATACTTTGATACCTCATCCTCCAT
SSADH2 reverse	GAGCAGCAGGATAGAAATTTGAATG
EF-1α forward	CTCCCACATTGCCGTCAAG
EF-1α reverse	GCCAGATCGCCTGTCGAT
AlaAT forward	CTGCAAAGACAGCCCCAGAT
AlaAT reverse	ACAACAACTCCTGTGGCATTGA
AMADH1 forward	AAAGGTGTGACCGTTTCTCTAAGG
AMADH1 reverse	AGCATGGTTGTGAGCAGTTGAT

AMADH2 forward	GCTGCAGTCATATCGAAAGATTTAGA
AMADH2 reverse	CCCGCTTGAAGGGCCTTA
AO1 forward	GACGTCCCTCCGAGCACAT
AO1 reverse	TGCAGCCATCCCGTTGT
AO2 forward	ACCAAAGCACAGGCGAAGAT
AO2 reverse	CGATTGGTCGGTCCCTGTT
AO3 forward	CCGAGGTGCATTTACCAATTACA
AO3 reverse	CCAGCCCATTTTTCAGACTTG
AO4 forward	AGTAGCCAAGCCGATTCAGAAC
AO4 reverse	GCTCGATCATACTAAAARATGATGC
AO5 forward	AGTAGCCAAGCCGATTCAGAAC
AO5 reverse	CAAGGTAGTCAAAGAAAGCATGTGTTT
PAO1 forward	GACGCTGTCTCAAGTCTCAAA
PAO1 reverse	TGCCTATAACTCCACCTAGTCA
PAO4 forward	CCCTCAGCATGCTTACCTTAT
PAO4 reverse	TCTTTCATGTCTGAAGTATCGGAA
PAO2 forward	TGAGAGTTCTTGAGCGTTATGG
PAO2 reverse	GAAGTTACAGGCGGGAGATAAG
PAO5 forward	AGCTACACACAGAACCCATTAC
PAO5 reverse	ATAAAGGTTCCAAAAATAGATAGAAAT
PAO6 forward	GCCAATAGGCTTCTCCAA
PAO6 reverse	GAAAGAGAAGCAAAGATGTTGA

Table S4. Impact of elevated CO_2 on the amino acid composition of 'Empire' apple fruit during 16 weeks of postharvest storage under low temperaure and low oxygen conditions. Data represent the mean ± SE (nmol per gram fresh mass) of four storage treatment replicates.

		Storage Period						
		Week 2		Week 4		Week 8		Week 16	
	Pre-Storage	0.03 kPa CO_2	5 kPa CO_2	0.03 kPa CO_2	5 kPa CO_2	0.03 kPa CO_2	5 kPa CO_2	0.03 kPa CO_2	5 kPa CO_2
Aspartate	563.1 ± 65.4	1206.0 ± 55.3	676.7 ± 96.0	1264.5 ± 61.2	763.4 ± 101.7	799.9 ± 96.0	426.4 ± 24.2	747.5 ± 63.4	686.5 ± 245.0
Glutamate	314.7 ± 23.0	854.1 ± 41.8	660.3 ± 41.3	803.6 ± 54.2	756.7 ± 77.1	438.0 ± 11.30	361.1 ± 28.9	381.0 ± 18.1	523.7 ± 241.2
Aspargine	895.5 ± 358.3	1006.1 ± 36.4	582.3 ± 175.5	798.8 ± 121.7	716.8 ± 95.0	772.6 ± 313.4	554.8 ± 252.3	473.3 ± 172.4	550.7 ± 130.3
Serine	197.8 ± 11.7	340.1 ± 21.3	339.3 ± 25.3	284.1 ± 20.7	305.4 ± 20.2	191.6 ± 12.4	160.8 ± 15.3	138.2 ± 7.6	193.9 ± 75.2
Glutamine	176.5 ± 33.9	154.5 ± 20.0	240.0 ± 30.1	87.1 ± 3.2	130.4 ± 11.0	50.9 ± 10.4	54.4 ± 21.8	54.9 ± 8.6	70.6 ± 16.6
Histidine	16.1 ± 0.4	27.4 ± 4.5	29.1 ± 1.5	26.0 ± 0.8	24.4 ± 0.4	18.9 ± 0.5	14.7 ± 1.6	15.1 ± 0.4	15.2 ± 2.3
Glycine	83.0 ± 3.1	94.7 ± 6.1	99.6 ± 8.7	85.2 ± 2.6	87.1 ± 4.4	100.5 ± 9.3	75.8 ± 2.5	63.5 ± 2.3	99.7 ± 45.8
Threonine	46.8 ± 3.3	57.8 ± 5.7	53.7 ± 4.4	58.6 ± 6.4	59.5 ± 4.2	48.0 ± 2.6	58.9 ± 3.1	28.0 ± 2.0	63.4 ± 21.5
Arginine	26.7 ± 2.2	78.7 ± 9.3	68.6 ± 6.7	63.7 ± 4.5	62.3 ± 2.8	35.3 ± 4.6	39.1 ± 2.7	26.6 ± 2.2	60.1 ± 30.5
Alanine	104.9 ± 13.8	292.1 ± 24.8	430.1 ± 72.8	231.0 ± 24.6	341.7 ± 54.3	119.2 ± 15.2	147.2 ± 47.1	94.2 ± 14.6	150.1 ± 39.3
GABA	71.5 ± 10.6	176.2 ± 14.6	162.3 ± 24.7	141.1 ± 6.2	199.0 ± 26.8	70.2 ± 9.5	130.4 ± 43.2	86.4 ± 7.6	238.8 ± 74.4
Tyrosine	8.8 ± 0.6	9.8 ± 0.9	13.6 ± 1.2	10.0 ± 0.6	11.5 ± 0.9	9.92 ± 0.9	9.2 ± 2.2	7.8 ± 0.4	13.5 ± 5.4
Cysteine	27.5 ± 2.7	30.4 ± 6.7	55.5 ± 21.9	31.5 ± 4.2	29.4 ± 3.6	30.2 ± 4.3	24.3 ± 3.0	23.1 ± 2.8	21.0 ± 4.7
Valine	7.9 ± 0.2	45.6 ± 2.9	35.9 ± 2.0	43.6 ± 2.4	35.6 ± 1.6	34.5 ± 3.1	22.0 ± 1.5	37.7 ± 1.9	41.8 ± 25.9
Methionine	24.9 ± 1.3	15.5 ± 1.9	13.6 ± 1.4	11.5 ± 0.7	10.4 ± 0.7	3.6 ± 0.5	3.5 ± 0.2	4.2 ± 0.3	13.4 ± 6.7
Phenylalanine	10.2 ± 0.9	15.0 ± 1.7	25.3 ± 3.0	15.0 ± 0.8	21.5 ± 1.9	10.0 ± 0.6	10.4 ± 3.3	6.4 ± 0.3	11.7 ± 5.5
Isoleucine	11.2 ± 0.6	17.3 ± 1.8	14.0 ± 1.0	18.0 ± 1.1	12.7 ± 0.8	23.9 ± 6.5	10.8 ± 0.7	26.8 ± 3.0	22.1 ± 11.4
Leucine	13.8 ± 0.8	22.6 ± 2.4	16.6 ± 1.9	20.6 ± 1.0	13.5 ± 1.3	21.3 ± 1.7	11.7 ± 0.6	18.2 ± 0.5	25.2 ± 14.0
Lysine	9.5 ± 1.2	21.4 ± 8.7	19.4 ± 4.8	11.6 ± 3.2	9.2 ± 2.2	1.4 ± 0.1	1.4 ± 0.1	8.7 ± 0.8	7.8 ± 2.4
Total amino acid	2610.3 ± 505.4	4465.3 ± 230.1	3535.8 ± 452.1	4005.4 ± 179.8	3590.5 ± 315.1	2779.6 ± 411.5	2116.6 ± 410.8	2241.4 ±298.7	1846.5 ± 429.5

Table S5. Percent global identity of known Arabidopsis and putative apple PAOs (created by Clustal 2.1)

	**AtPAO5**	**MdPAO5**	**MdPAO6**	**AtPAO1**	**MdPAO1**	**MdPAO2**	**AtPAO2**	**AtPAO3**	**AtPAO4**	**MdPAO4**	**MdPAO3**
**AtPAO5**	100.0										
**MdPAO5**	61.38	100.0									
**MdPAO6**	61.19	90.48	100.0								
**AtPAO1**	24.03	24.76	24.11	100.0							
**MdPAO1**	22.78	25.53	24.36	72.46	100.0						
**MdPAO2**	27.38	28.14	28.87	27.84	29.19	100.0					
**AtPAO2**	26.73	27.51	27.31	29.40	29.86	79.14	100.0				
**AtPAO3**	25.95	26.98	27.02	27.74	28.18	78.44	85.42	100.0			
**AtPAO4**	27.55	29.00	28.51	29.22	29.62	59.80	61.84	61.07	100.0		
**MdPAO4**	26.43	27.44	27.42	27.36	27.13	60.16	62.22	60.82	67.55	100.0	
**MdPAO3**	26.19	26.98	26.96	27.65	27.42	60.91	61.93	60.33	67.28	91.67	100.0

Supplementary Figures

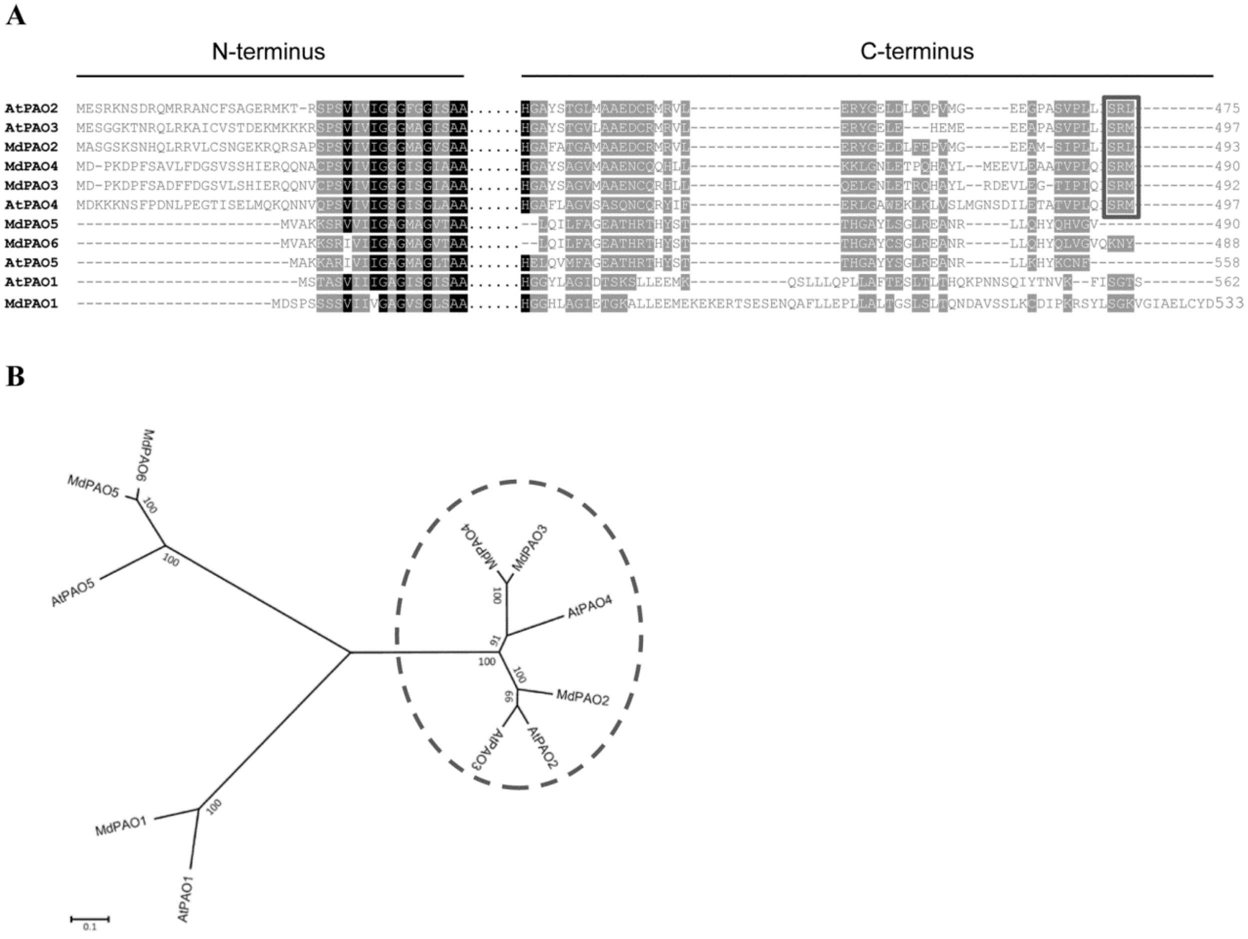

Figure S1. (A) Multiple amino acid sequence alignment of the N- and C-termini of apple and Arabidopsis PAOs. Sequence alignment was performed with the CLUSTALW2 software[4] and edited manually. Number of total amino acids is shown on the right. The presence of C-terminal peroxisomal targeting signals is indicated by the black box. Identical residues are shown with a black background, and similar residues are shown with a grey background. (B) Phylogenetic relationship of PAO members in apple and Arabidopsis. The unrooted neighbor-joining method was utilized to perform the phylogenetic analysis. The Phylogram was created by ClustalW2 multiple sequence alignment and MEGA 6.0 software. Proteins carrying putative peroxisome

targeting signals are circled by a dashed line. Internal numbers give bootstrap frequencies for each clade.

REERENCES

1 Skoog DA, Holler FJ, Nieman TA. *Principles of Instrumental Analysis, Fifth Edition.* Harcourt Brace & Company, New York, USA. 1998; Pp. 18.

2 Queval G, Noctor G. A plate reader method for the measurement of NAD, NADP, glutathione, and ascorbate in tissue extracts: Application to redox profiling during Arabidopsis rosette development. *Analyt Biochem* 2007; **363:** 58–69.

3 Lum GB, Brikis CJ, Deyman KL, Subedi S, DeEll JR, Shelp BJ, Bozzo GG. Pre-storage conditioning ameliorates the negative impact of 1-methylcyclopropene on physiological injury and modifies the response of antioxidants and γ-aminobutyrate in 'Honeycrisp' apples exposed to controlled-atmosphere conditions. *Postharv Biol Technol* 2016; **116:** 115-128.

4 Larkin MA, Blackshields G, Brown NP, Chenna R, McGettigan PA, McWilliam H *et al.* Clustal W and Clustal X version 2.0. *Bioinformatics* 2007; **21:** 2947-2948.
